# Standardization of radiation therapy quality control system through mutual quality control based on failure mode and effects analysis

**DOI:** 10.1007/s12194-024-00857-z

**Published:** 2024-11-18

**Authors:** Yuki Tanimoto, Masataka Oita, Kazunobu Koshi, Kiyoshi Ishiwaki, Futoshi Hiramatsu, Toshihisa Sasaki, Hiroki Ise, Takashi Miyagawa, Takeshi Maeda, Shinsuke Okahira, Takashi Hamaguchi, Tatsuya Kawaguchi, Norihiro Funada, Shuhei Yamamoto, Akira Hiroshige, Yuki Mukai, Shohei Yoshida, Yoshiki Fujita, Atsuki Nakahira, Hirofumi Honda

**Affiliations:** 1 Department of Radiology, NHO Kure Medical Center and Chugoku Cancer Center, Kure, 737-0023 Japan; 2 Graduate School of Interdisciplinary Science and Engineering in Health Systems, Okayama University, Okayama, 700-8558 Japan; 3 Faculty of Interdisciplinary Science and Engineering in Health Systems, Department of Healthcare Science, Okayama University, 3-1-1 Tsushima-Naka, Kita-Ku, Okayama, 700-8530 Japan; 4 Department of Radiology, NHO Fukuyama Medical Center, Fukuyama, 720-8520 Japan; 5 Department of Radiology, NHO Iwakuni Medical Center, Iwakuni, 740-8510 Japan; 6 Department of Radiology, NHO Hamada Medical Center, Hamada, 697-8511 Japan; 7 Department of Radiology, NHO Higashi-Hiroshima Medical Center, Higashi-Hiroshima, 739-0041 Japan; 8 Department of Radiology, NHO Kanmon Medical Center, Shimonoseki, 752-8510 Japan; 9 Department of Radiology, NHO Kochi National Hospital, Kochi, 780-8507 Japan; 10 Department of Radiology, NHO Yamaguchi-Ube Medical Center, Ube, 755-0241 Japan; 11 Department of Radiology, NHO Okayama Medical Center, Okayama, 701-1192 Japan; 12 Department of Radiology, NHO Shikoku Medical Center for Children and Adults, Zentsuji, 765-8507 Japan; 13 Department of Radiology, NHO Shikoku Cancer Center, Matsuyama, 791-0280 Japan; 14 Department of Radiological Technology, Ehime University Hospital, Matsuyama, 791-0295 Japan

**Keywords:** Radiation therapy, Quality control, Failure mode and effects analysis, Cost-effectiveness

## Abstract

The advancement of irradiation technology has increased the demand for quality control of radiation therapy equipment. Consequently, the number of quality control items and required personnel have also increased. However, differences in the proportion of qualified personnel to irradiation techniques have caused bias in quality control systems among institutions. To standardize the quality across institutions, researchers should conduct mutual quality control by analyzing the quality control data of one institution at another institution and comparing the results with those of their own institutions. This study uses failure mode and effects analysis (FMEA) to identify potential risks in 12 radiation therapy institutions, compares the results before and after implementation of mutual quality control, and examines the utility of mutual quality control in risk reduction. Furthermore, a cost-effectiveness factor is introduced into FMEA to evaluate the utility of mutual quality control.

**Supplementary Information:**

The online version contains supplementary material available at 10.1007/s12194-024-00857-z.

## Introduction

High-precision radiation therapy modalities, such as intensity-modulated radiation therapy (IMRT), stereotactic radiosurgery, and stereotactic body radiation therapy, are more sensitive to instrumentation errors than three-dimensional conformal radiation therapy (3D-CRT). Therefore, stringent tolerances have been established in various guidelines [1–4]. In addition, with the introduction of volumetric modulated arc therapy and an increase in the number of quality control items, the burden on radiation therapy equipment’s quality control personnel has increased. Several radiation therapy-related accidents caused by incorrect entry or poor communication have been reported [5]. The Japanese Society of Radiation Oncology (JASTRO) recommends output dose evaluation by a third-party output-evaluation organization at least once every three years. Accordingly, various evaluation methods have been reported [6–8]. However, in our hospital group, differences in the number of qualified personnel and the irradiation techniques employed have resulted in differences in the quality control systems of radiation therapy equipment. Therefore, we believe more parameters than the ones proposed by the JASTRO require quality control.

To standardize the quality control system within our hospital group, we aimed to establish a mutual quality control system in which data analysis related to the quality control of our own institution is conducted in other institutions, and the results were compared with those of our institutions [9]. However, because peer quality control is not based on risk analysis, it cannot sufficiently address the potential risks *from the introduction of equipment to the start of treatment* and *daily quality control*. According to the American Association of Physicists in Medicine Task Group-100 (AAPM TG-100), most analyses performed in the field of radiation therapy are based on failure mode and effects analysis (FMEA) [10–15].

Amit et al. developed a quality assurance (QA) method for dynamic multi-leaf collimators based on FMEA [16]. In addition, Jacqueline et al. studied specific QA items for IMRT indicated in TG-40 and TG-142 by requesting physicists in different regions to perform FMEA to identify the risks associated with these items [17–19]. Jennifer et al. used FMEA to determine the frequency of QA testing based on TG-142 [20].

Mutual quality control requires analysis of data from other institutions in addition to the typical work. Per the Labor Standards law in Japan, we assumed that an institution would pay its employee overtime (overtime work) for support work. Therefore, the utility of mutual quality control among institutions must be proven, considering the cost of overtime (cost-effectiveness), which would not have occurred in the case of quality control at a single institution. Zhengzheng et al. incorporated the concept of cost into FMEA in the field of radiation therapy [21]. An FMEA of delays in the period from treatment planning CT to the start of treatment was conducted, and an index of socioeconomic impact named “RPNSE-EE” was proposed and evaluated. However, few studies on FMEA and cost in the day-to-day quality control of radiation therapy and few reports on the cost-effectiveness of insurance reimbursement in Japan have been published.

The aims of this study are 1) to identify potential risks using FMEA, compare the results of risk analysis before and after the implementation of mutual quality control, and verify the utility of mutual quality control in risk reduction; and 2) to calculate FMEA_cost-eff_ and then re-examine the utility of mutual quality control considering cost-effectiveness.

## Materials and methods

### Failure modes and effect analysis (FMEA) structure and objectives

A process map for the quality control of equipment—from commissioning of the linear accelerator to irradiation of patients—was developed by six representatives from five institutions who were certified as either radiation therapy technologists or medical physicists. Subsequently, the failure modes in each process step were identified by the representatives. We requested the representatives to identify at least one mode per institution, as recommended in previous studies [22].

As this study is related to the quality control of equipment, physicians or nurses may lack the expertise required numerical analysis. Therefore, the identification of failure modes was limited to a list of items related to the quality control of equipment performed by radiological technologists or medical physicists.

Three indices, namely occurrence (O), severity (S), and detectability (D), were set for each failure mode with reference to TG-100 [15]. Each category was scored on a 10-point scale, with 10 being the highest probability of occurrence, severity, and difficulty of detection. The risk priority number (RPN) was calculated by multiplying the three parameters.

The FMEA analysis was evaluated based on the quality control system of our institution, including staffing, quality control items, and measurement frequency, for each failure mode. To prevent significant numerical bias for each parameter, a severity (S) parameter for each failure mode was defined separately for high- and non-high-precision radiation therapy institutions (3D-CRT institutions) by a representative. Detectability (D) was defined by this representative with reference to the literature on the usefulness of double-checking and independent double-checking for detectability (Table 1) [23, 24]. Occurrence (O) rates were defined with reference to TG-100 (Table 2) [15]. Analysts may choose occurrence (O), severity (S), and detectability (D) depending on the operational status of their institutions. After removing the significant outliers in the first evaluation and publishing the results of the second one, another evaluation was requested. The recurring significant outliers were individually interviewed [25, 26]. One of the purposes of the first round of FMEA was to correct ambiguous or inaccurate wordings in the failure modes. The second round of FMEA was performed with the addition of an explanation of the relevant section. The interviews were conducted to ascertain the presence of operational ambiguities at the concerned institutions. However, the interviews were conducted after reiterating that the main objective was to reflect the situation at each institution. It was analyzed by 13 persons from 12 institutions, including 6 representatives. The average of 12 institutions for each disability mode was calculated. The targets for mutual quality control were failure modes with RPN average values in the top 20% and a quality control that could be performed by a third party through posterior analysis, referring to AAPM TG-100 [15].

**Table 1:** Detectability (D) as defined by the representative

Rank	Combination of detectability	
1	Analysis by a qualified personnel	Independent double-checking by a qualified personnel
2	Analysis by a qualified or an unqualified personnel	Independent double-checking by a qualified personnel
3	Analysis by a qualified personnel	Double-check by a qualified personnel
4	Analysis by a qualified or an unqualified personnel	Double-check by a qualified personnel
5	Analysis by an unqualified personnel	Double-check by a qualified personnel
6	Analysis by an unqualified personnel	Double-check by a qualified or an unqualified personnel
7	Analysis by an qualified personnel	–
8	Analysis by a qualified or an unqualified personnel	–
9	Analysis by an unqualified personnel	–
10	Non-analysis	–

**Table 2:** Occurrence defined with reference to TG-100

Rank	Occurrence	
	Qualitative	Frequency in %
1	Failure unlikely	0.01
2		0.02
3	Relatively few failures	0.05
4		0.1
5		< 0.2
6	Occasional failures	< 0.5
7		< 1
8	Repeated failures	< 2
9		< 5
10	Inevitable failures	> 5

### Data analysis

The obtained RPNs were compared using EZR (Saitama Medical Center, Jichi Medical University, Saitama, Japan), which is a graphical user interface of R (The R Foundation for Statistical Computing). The Mann–Whitney *U* test [22, 27] was conducted to compare the differences in irradiation techniques between high-precision radiation therapy and 3D-CRT, as well as the number of qualified personnel per linear accelerator and the number of personnel with over 10 years of experience per linear accelerator. Comparisons of the number of qualified personnel and those with over 10 years of experience were made based on whether there were at least two persons or at most one person per linear accelerator, with “at most one” indicating the absence of such personnel. The RPN was considered statistically significant if *p* ≤ 0.05. The number of subjects from the participating institutions under each condition is listed in Table 3.

**Table 3:** Number of persons in each condition at participating institutions

	≥ 2	≤ 1
10 years of experience per linear accelerator	6	6
Number of qualified personnel per linear accelerator	7	5
	High-precision radiation therapy	3D-CRT
Irradiation technique	6	6

### Determination of mutual quality control methods

**Fig. 1:**
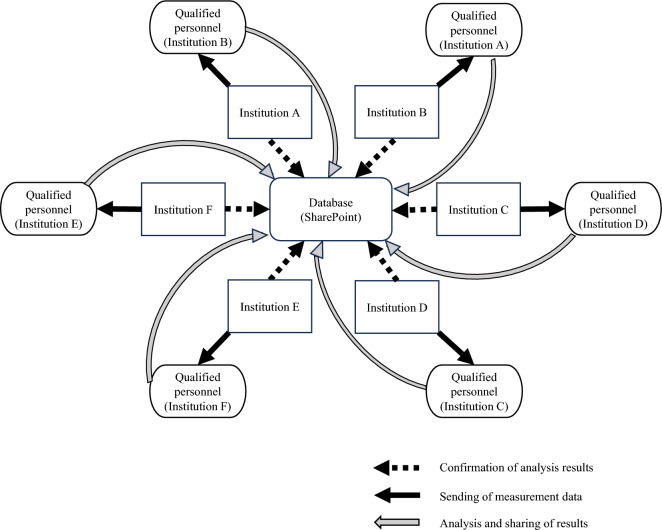
Mutual quality control overview

Based on the results of Sect. 2.2, a method of mutual quality control was devised by the representatives (Fig. 1). The RPN before and after the mutual quality control were compared to verify the effectiveness of mutual quality control. The differences in RPN depending on the number of qualified personnel per linear accelerator were also compared.

### Calculation of FMEA_cost-eff_

FMEA_cost-eff_ was calculated to identify the cost-effectiveness of mutual quality control. FMEA_cost-eff_ was calculated from Eq. (1) with reference to the incremental cost-effectiveness ratio (ICER) [28].

1 $${FMEA}_{cost-eff}=\frac{{C}_{B}-{C}_{A}}{{RPN}_{A}-{RPN}_{B}},$$

where *C*_*A*_ and *C*_*B*_ and *RPN*_*A*_ and *RPN*_*B*_ are the costs and RPN before and after mutual quality control, respectively.

The cost was based on a predetermined salary of *¥342,500* for radiological technologists at all company sizes (10 or more employees) from “2021 Basic Survey on Wage Structure” published by the Ministry of Health, Labor and Welfare [29]. This predetermined salary was converted into actual working hours (162 h) and then into hourly wage with reference to the Labor Standards Law in Japan, and finally multiplied by 1.25 to calculate the hourly wage for overtime work. Meanwhile, *C*_*B*_ was calculated by multiplying the time spent by the three mutual quality performers on the three times mutual quality control by the hourly rate for overtime work and adding it to *C*_*A*_.

## Results

### Identification of high risk factors by FMEA

**Fig. 2:**
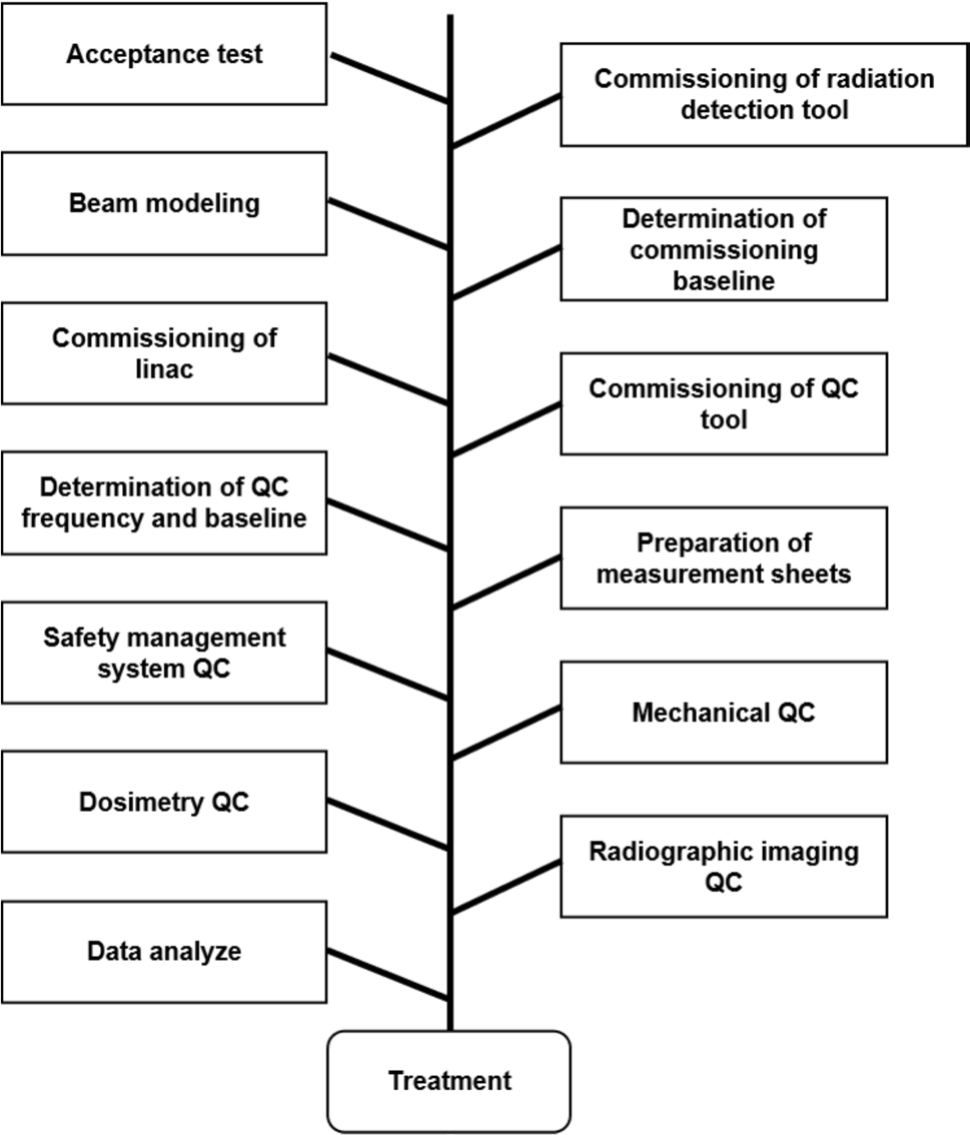
Process map of *from the introduction of the equipment to the start of treatment* and *daily quality control*

**Fig. 3:**
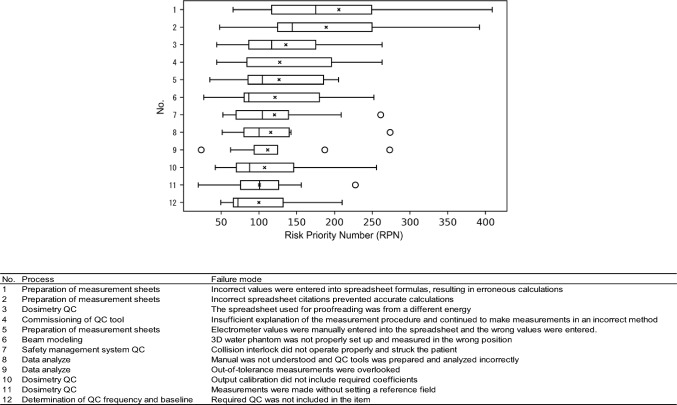
Box plot representation of the mean risk priority number (RPN) scores of the top 20% failure modes

**Fig. 4:**
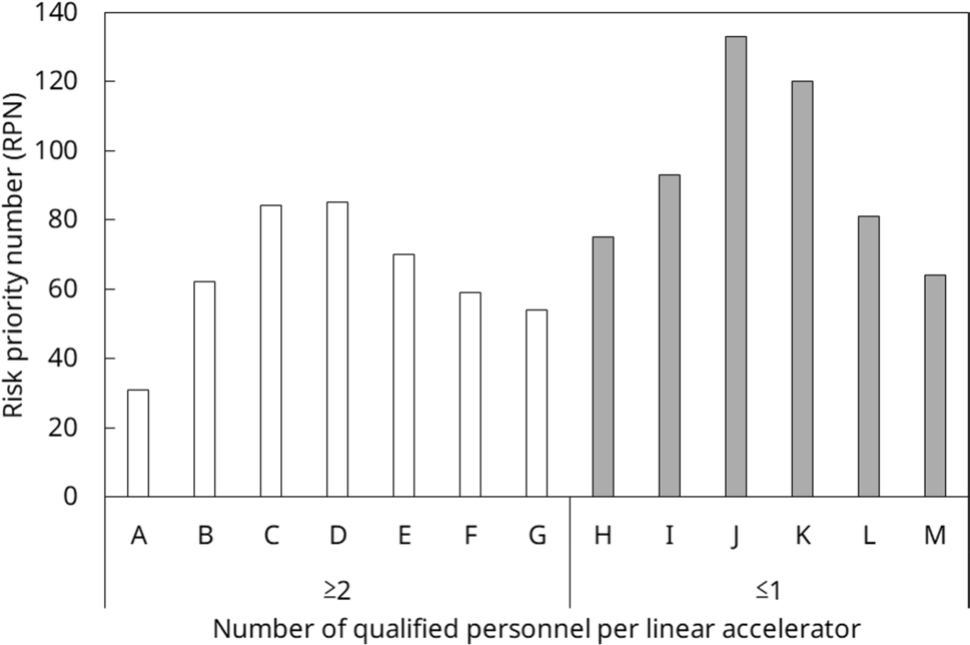
Mean RPN, grouped by the number of qualified personnel per linear accelerator

Process maps were created for 14 processes (Fig. 2). Sixty failure modes were identified for all processes, and the top 20% of RPN averages are illustrated in Fig. 3. The number of identified failure modes averaged 12 per institution, with the lowest being 4 per institution. The Mann–Whitney *U* test revealed that the mean values for high-precision irradiation and 3D-CRT were higher at institutions that performed high-precision irradiation. Nevertheless, the difference was not statistically significant (*p* = 0.068). Moreover, there was no difference in the RPN between institutions with one person with ten or more years of treatment experience per linear accelerator and those with at least one person with ten or more years of experience per linear accelerator (*p* = 0.223). However, when there were two or more qualified personnel per linear accelerator, the RPN was statistically lower than when there were less than one (*p* = 0.0256) (Fig. 4, Online Resource 2).

### Changes in RPN after mutual quality control

**Fig. 5:**
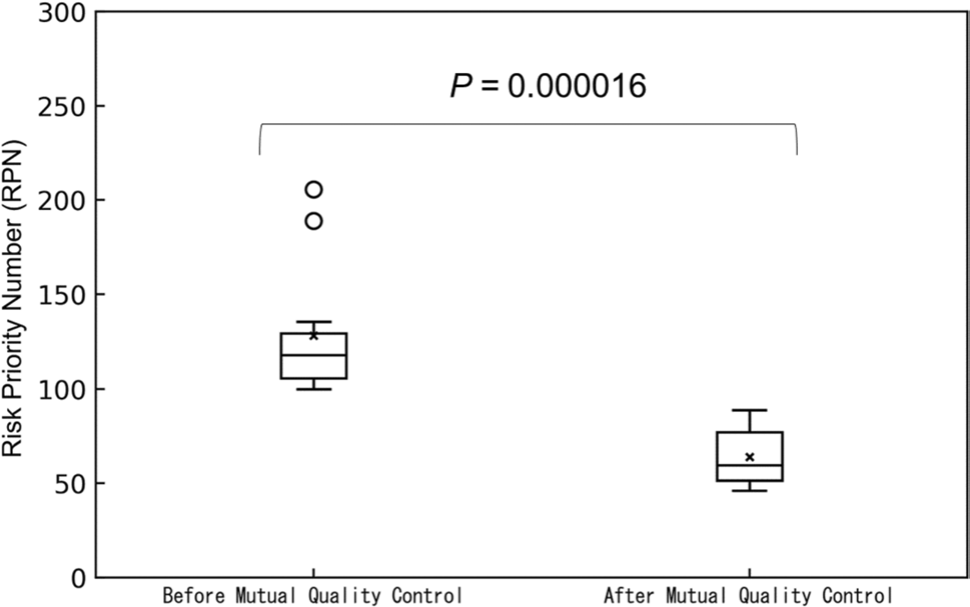
Change in RPN before and after implementation of mutual quality control. The average RPN values of the 13 FMEA analysts for each failure mode are shown

The changes in FMEA arising from the implementation of the mutual quality control items identified in Sect. 3.1 was re-evaluated at 12 institutions, with an average RPN of 64.0 ± 15.4. This was a significant decrease from the mean value of 128.3 ± 34.4 before the implementation of mutual quality control (Fig. 5).

**Fig. 6:**
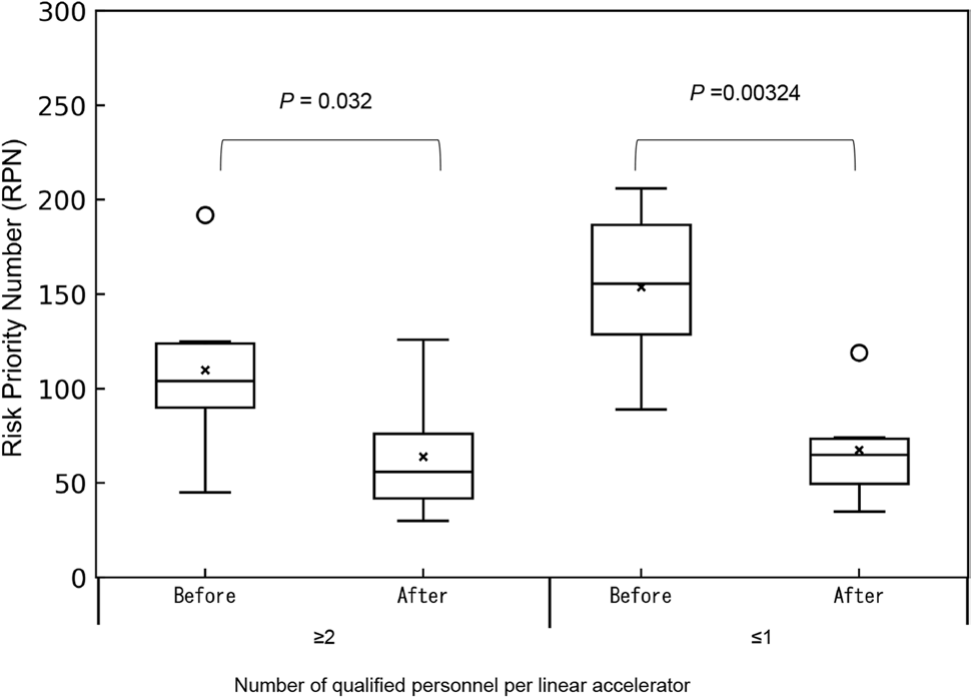
Change in RPN before and after mutual quality control by number of qualified personnel per linear accelerator. The average RPN values of the failure modes for each of the 13 FMEA analysts are shown

For example, in “[Beam modeling] 3D water phantom was not properly set up and measured in the wrong position,” a lack of standardized procedures and inadequate training were considered as probable causes of this decrease. As a countermeasure, beam data were published on a database and compared with those from other institutions. In addition, data were analyzed using PyMedPhys by qualified personnel. As illustrated in Fig. 6, the RPN decreased with the number of qualified personnel per linear accelerator. Additionally, a statistically significant difference was observed.

### Costs required for risk reduction

The average time required for mutual quality control was 46.6 ± 4.4 min. According to the 2021 Basic Survey on Wage Structure, the salary of medical radiological technologists for the total company size (10 or more employees) was *¥342,500* [29]. The cost of mutual quality control was *¥2,053.4* per month; hence, *C*_*A*_ was *¥0*, *C*_*B*_ was *¥2,053.4*, *RPN*_*A*_ was *128.3*, and *RPN*_*B*_ was *64.0*. Based on these values, the FMEA_cost-eff_ was calculated to be *¥31.9/RPN* [Online Resource 2].

## Discussion

### Evaluation of RPN

The minimum number of failure modes identified by the representatives was 4, and the total number of failure modes was 60. The number of failure modes was discussed among the representatives and deemed to be valid. As mentioned earlier, this study identified the utility of mutual quality control for risk reduction in the quality control of radiation therapy equipment. The top 20% RPN average failure modes were monitor chamber calibration, beam profile measurement, and QA data analysis. Bruce et al. stated that most events in radiation therapy occurred due to human error and not equipment failure, and that the risks cited in their study may also be induced by human errors due to lack of training or poor software design [30]. The fact that there was no significant difference in the RPN value by the irradiation technique suggests that the RPN is not only necessary for institutions that provide high-precision radiation therapy, but also for those that provide only 3D-CRT. The results also suggest that possession of certification and not experience was useful in risk reduction. Such certification requires extensive knowledge to obtain. The study is consistent with previous reports in the field of radiology regarding years of experience and occurrence of incidents and other risks, with no relationship between years of experience and the number of incidents [31].

Based on these results, a verification sheet of the calibration results of monitor dosimeters should be prepared. Quality control of TG-142 using Pylinac and gamma analysis of dose profiles using PyMedPhys should be performed with the results of the analysis hidden. Mutual quality controls should be performed by qualified personnel from other institutions [32, 33]. One factor contributing to the reduction in RPN before and after mutual quality control was the use of Microsoft SharePoint (Microsoft Corporation, USA) to output results. This allowed for the comparison of results from multiple institutions and our own institution's results, which were considered to have led to a reduction in risk (Fig. 4). It should be noted that Nishioka et al. have proposed a method for collaborative online working on FMEA worksheets, which might suggest to improve risk management efficiency through enhanced data sharing [34]. A schematic of mutual quality control is depicted in Fig. 1.

The RPN values significantly decreased before and after implementation of mutual quality control. In quality control operations, the typical methods to reduce risk are to purchase new quality control tools or supplement them with new experienced personnel. The effectiveness of double-checking by qualified personnel has also been described in previous studies [24]. Mutual quality control in this study also enabled double-checking by qualified personnel, which was considered an effective countermeasure against human error. Toyama et al. discovered that medical physicists employed by radiology technologists spent a significant amount of their time on patient irradiation and imaging work, and that 49.7% of them worked overtime on duty. This indicates that there is still no full-time radiation therapy quality control staff, and that the increased burden on qualified staff has not been resolved [35].

However, even for institutions where implementation is difficult due to cost and a lack of human resources, acceptable results can be obtained at a low cost through mutual quality control, and the cost required for risk reduction was determined using FMEA_cost-eff_.

### Limitations and future issues

A limitation of this study is the small sample size. Although we were able to include all radiation therapy equipment-owning institutions of the group in the analysis, further studies should increase the number of institutions and examine the resulting effects of mutual quality control. Although this method does not support patient monitoring, the results of the analysis can be received remotely by an experienced and qualified person simply by transmitting the analysis data to their systems. The disclosure of analysis results and sharing of information have the advantages of eliminating black-boxing and clarifying device-specific deviations, which can be used as references in determining the allowable values.

## Conclusion

The utility of mutual quality control of radiation therapy equipment was examined using FMEA considering the cost-effectiveness. Mutual quality control is useful for risk reduction, and FMEA_cost-eff_ can determine the cost required for risk reduction, enabling us to judge its usefulness after considering the cost. Currently, per the requirements for a medical institution to be designated as a Regional Base Hospital for Cancer Treatment, the quality control of radiation therapy equipment should be performed by a person with a professional qualification in medical physics. The need for specialized qualifications is increasing; however, recruiting more qualified personnel is difficult. We believe that this study will help increase this number [36].

## Supplementary Information

Below is the link to the electronic supplementary material.

Supplementary file1 (XLSX 30 KB)

Supplementary file2 (XLSX 18 KB)

## Data Availability

The authors declare that the data supporting the findings of this study are available within the paper. Data also will be made available on reasonable request.
